# The Association Between Hair Loss and COVID-19: The Impact of Hair Loss After COVID-19 Infection on the Quality of Life Among Residents in Saudi Arabia

**DOI:** 10.7759/cureus.30266

**Published:** 2022-10-13

**Authors:** Salim Alkeraye, Anwar Alrashidi, Noura S Alotaibi, Norah Almajli, Bashayr Alkhalifah, Norah Bajunaid, Raghad Alharthi, Tuqa AlKaff, Koloud Alharbi

**Affiliations:** 1 Department of Dermatology, King Saud University, College of Medicine, Riyadh, SAU; 2 College of Medicine, Princess Nourah Bint Abdulrahman University, Riyadh, SAU; 3 Dermatology, King Abdulaziz Medical City Riyadh, Riyadh, SAU; 4 Dermatology, King Khalid University Hospital, Riyadh, SAU; 5 Dermatology, Security Forces Hospital, Riyadh, SAU

**Keywords:** saudi arabia, telogen effluvium, hair loss, impact, prevalence, covid-19

## Abstract

Background

Coronavirus disease 2019 (COVID-19) is an infectious disease that is associated with many health conditions, including, but not limited to, dermatological diseases. Some patients suffer from hair loss after becoming infected with severe acute respiratory syndrome coronavirus 2. Acute telogen effluvium (TE) is a non-scarring hair loss that usually occurs three months after a stressful event and can last up to six months, and it can be associated with post-COVID-19 infections. This study aims to explore the prevalence of hair loss among recovered COVID-19 patients in Saudi Arabia and determine the contributing factors. Furthermore, we aimed to measure the impact of hair loss after COVID-19 on their quality of life.

Methodology

An observational, cross-sectional study was conducted from September to December 2021 in Saudi Arabia. A questionnaire was used to assess hair loss after being infected with COVID-19. Furthermore, quality of life was assessed using the Dermatology Life Quality Index (DLQI) questionnaire. Participants were recruited by sharing the questionnaire on social media platforms (Twitter, Telegram, and WhatsApp). The data were analyzed using SPSS version 23 (IBM Corp., Armonk, NY, USA). Frequency and percentages were used to display categorical variables, while minimum, maximum, mean, and standard deviation were used to present continuous variables. The categorical variables were compared using a chi-square test, and the statistical significance cut-off was set at p < 0.05.

Results

A total of 806 participants were included in the study, of whom 52.7% experienced hair loss after COVID-19 infection. Age, gender, high temperature during, and the presence of hair loss prior to infection were significantly associated with the incidence of TE. The severity of life affection based on DLQI showed that 91.4% of the participants did not have severe life affection, while 8.6% had their lives severely affected.

Conclusions

This study revealed subjective hair loss that was significantly associated with high temperature, being female, and having a history of previous hair loss. Further studies using objective assessments are suggested for reaching more precise conclusions.

## Introduction

The novel coronavirus disease 2019 (COVID-19) outbreak was declared a global pandemic by the World Health Organization (WHO) on March 11, 2020 [1]. COVID-19 has infected more than 76 million persons over the last year and caused more than 3.8 million deaths worldwide [2].

A variety of skin manifestations in COVID-19 patients have been reported. According to a case series from an international registry from the American Academy of Dermatology and the International League of Dermatological Societies, a morbilliform rash was the most commonly reported dermatological finding in laboratory-confirmed COVID-19 patients [3]. In addition, various studies have shown that some patients developed hair loss after severe acute respiratory syndrome coronavirus 2 (SARS-CoV-2) infections [4]. A related prospective study conducted at Istanbul Medeniyet University revealed that out of 75 (36.8%) telogen effluvium (TE) cases, 57 (27.9%) were considered COVID-19-associated TE [5].

TE is the most common cause of non-scarring alopecia. It is characterized by non-scarring, diffuse hair loss from the scalp. It occurs as a reaction to various insults that can be physical, mental, or chemical, resulting in a premature follicular transition from the anagen (active growth phase) to the telogen (resting phase). Acute TE is a self-limiting disorder that usually lasts for about six months, after which it usually goes into remission [6].

A large longitudinal study on 538 COVID-19 survivors and 184 controls was conducted in Wuhan, China, to investigate the prevalence and predictors of COVID-19 clinical sequelae [4]. Alopecia was among the most prevalent complaint in convalescent COVID-19 patients, occurring after three to four months and being reported more commonly by women [4]. Multiple studies have been conducted to understand the association between COVID-19 infections and the development of TE; however, evidence concerning the prevalence of TE among recovered COVID-19 patients in Saudi Arabia is currently limited. Hence, our study aims to explore the prevalence of hair loss among recovered COVID-19 patients and measure the impact of hair loss after COVID-19 on the quality of life among residents of Saudi Arabia.

## Materials and methods

This observational, cross-sectional study was conducted from September to December 2021 in Saudi Arabia. An online questionnaire was created using Google Forms and distributed on social media platforms (Twitter, Telegram, and WhatsApp). All adult patients residing in Saudi Arabia who were previously diagnosed with COVID-19 using either polymerase either chain reaction (PCR) or antibody testing, had experienced hair loss, and consented to participate in the study were included. Respondents with a family history of alopecia were also included in this study to prove whether COVID-19 activates alopecia in people who are genetically prone to androgenic alopecia. Subjects without a history of COVID-19 or with a history of COVID-19 but without a nasopharyngeal swab or hematological assessment were excluded. Participants who had experienced stressful events; had any of the following diseases: anemia, polycystic ovary syndrome, autoimmune diseases, thyroid disorder, and other endocrine disorders; and/or had recently used one of the following medications: anti-dihydrotestosterone, oral contraceptives, testosterone, anticoagulants, chemotherapeutic agents, and acne, cholesterol-lowering, anti-hypertensive, depression, bipolar, and gout medications were not included in the analysis, as those factors might cause hair loss. The study was approved by the Institutional Review Board at King Saud University (E-21-6483).

The impact of hair loss on the quality of life was assessed using the Arabic version of the Dermatology Life Quality Index (DLQI) questionnaire. This questionnaire is self-explanatory and can be simply filled out by the respondents in one or two minutes. There are 10 questions in the DLQI designed to asses symptoms, feelings, personal relationships, leisure, work, school, daily activities, and treatment. Each question has four possible answers “very much” (score 3), “a lot” (score 2), “a little” (score 1), and “not at all” (score 0). “Not relevant” is also scored as 0. The DLQI score is then calculated by summing the score of each question resulting in a maximum score of 30 and a minimum score of 0. The higher the score, the more quality of life is impaired. According to the score of the DLQI, the impact of hair loss on the quality of life is then graded as no effect at all (0-1), small effect (2-5), moderate effect (6-10), very large effect (11-20), and extremely large effect (21-30) on the patient’s life.

The study was performed using a convenience random sampling technique. The calculated sample size was 385 using the formula n = z2pq\d2 (n = sample size, z = z-score for 95% confidence interval, p = estimated proportion, q = p-1, d = margin of error) with a confidence level of 95%, an estimated proportion of 50%, and a 5% level of precision. A total of 2,500 COVID-19 laboratory-confirmed or suspected volunteers from all regions of Saudi Arabia filled out the questionnaire. However, 1,694 were excluded due to a lack of diagnosed infection by nasopharyngeal swab or hematological assessment. In total, 806 participants enrolled in this study, which was higher than the calculated minimum sample size and should thus increase the reliability of the study’s findings and ensure the sufficiency and accuracy of the findings.

The following factors were used in the prediction model: age, gender, marital status, place of residence, experiencing a rise in temperature associated with COVID-19, being hospitalized due to COVID-19, being admitted to the intensive care unit (ICU) due to COVID-19, having a history of hair loss before having COVID-19, and having a positive family history of alopecia. The data were analyzed using SPSS version 23 (IBM Corp., Armonk, NY, USA). Frequency and percentages were used to display categorical variables, while minimum, maximum, mean, and standard deviation were used to present continuous variables. The categorical variables were compared using a chi-square test, and the statistical significance cut-off was set at p < 0.05. Multivariate logistic regression was utilized to predict the risk factors for hair loss post-COVID-19 infection.

## Results

Among the 806 subjects diagnosed with COVID-19, 490 (60.8%) were females, and 491 (60.9%) were single. The majority (212 (26.3%)) were from the western region, Overall, 400 (49.6%) were 15-25 years old, followed by 209 (25.9%) who were 26-35 years old (Table 1). COVID-19 was diagnosed through PCR swabs in the mouth or nose in 99.55% of the patients, with only 0.5% being diagnosed by a blood test (antibodies) (Table 2).

**Table 1:** Sociodemographic profile of the participants (n = 806).

Demographical characteristics	n	%
Age		
15–25 years	400	49.60
26–35 years	209	25.90
36–45 years	126	15.60
46 years and older	71	8.80
Gender		
Male	316	39.20
Female	490	60.80
Marital status		
Single	491	60.90
Married	294	36.50
Divorced	15	1.90
Widowed	6	0.70
Place of residency		
Eastern region	179	22.20
Central region	172	21.30
Western region	212	26.30
Southern region	147	18.20
Northern region	96	11.90

**Table 2:** Participants’ COVID-19 infection profile (n = 806).

Question	n	%
Q1: How was it confirmed that you had COVID-19?		
Test by swab in the mouth or nose	802	99.5
Blood test (antibodies)	4	0.5
Q2: Did your temperature rise while you were infected with COVID-19?		
Yes	554	68.7
No	252	31.3
Q3: While you were infected with COVID-19, have you been hospitalized?		
Yes	84	10.4
No	722	89.6
Q4: During your COVID-19 infection, have you been admitted to the intensive care unit?		
Yes	6	0.7
No	800	99.3

After being infected with COVID-19, 425 (52.7%) participants had experienced hair loss, 243 (30.1%) had not, and 138 (17.1%) were not sure if they had. Scalp symptoms associated with alopecia in patients with COVID-19 were dandruff (19%), itching (17.5%), pain (8.4%), and a burning sensation (3.5%); 64.4% of the study’s participants did not experience any scalp symptoms.

Of the participants, 196 (46.12%) noticed hair loss one to three months after having COVID-19, followed by 119 (28%) who noticed it one week or less after contracting COVID-19. A total of 140 (32.94%) participants suffered hair loss for more than six months after being infected with COVID-19, while in 111 (26.12%) participants, hair loss lasted one to three months. In response to questions about hair loss complaints pre-pandemic and a family history of alopecia, 426 (52.9%) and 539 (66.9%) participants reported not having previous hair loss or a family history of alopecia, respectively. The majority (86%) of the participants who experienced alopecia had not consulted a dermatologist for this problem (Table 3).

**Table 3:** Participants’ hair loss profile.

Question	n	%
Q1: If your answer was yes, how long did your hair loss last? (n = 425)		
1 week or less	35	8.24
1–3 months	111	26.12
4–6 months	55	12.94
More than 6 months	140	32.94
I’m not sure	84	19.76
Q2: When did you notice your hair loss after contracting COVID-19? (n = 425)		
1 week or less	119	28.00
1–3 months	196	46.12
4–6 months	24	5.65
More than 6 months	12	2.82
I’m not sure	74	17.41
Q3: Have you consulted a dermatologist/doctor about your hair loss? (n = 425)		
Yes	57	13.41
No	368	86.59
Q4: Had you ever suffered from hair loss before contracting COVID-19? (n = 806)		
Yes	380	47.1
No	426	52.9
Q5: Do you have a family history of alopecia? (n = 806)		
Yes	178	22.1
No	539	66.9
I’m not sure	89	11

There was a significant association of age (p = 0.003), gender (p < 0.001), high temperature during COVID-19 infection (p = 0.002) ,and having a previous history of hair loss before COVID-19 (p = 0.002) with the incidence of hair loss post-COVID-19 (p < 0.001). Those who were 46 years and older had a notably lower rate of hair loss compared to the other age groups, whereas females had a notably higher rate of hair loss compared to males (75.6% vs 45%) (Table 4). To predict the risk factors for the incidence of hair loss post-COVID-19, multivariate logistic regression was used, and the following factors were attributed to a higher incidence of hair shedding after COVID-19: living in the western region (p = 0.002, odds ratio = 2.32), living in the southern region (p = 0.019, odds ratio = 2.06), and having a high temperature when infected with COVID-19 (p = 0.001, odds ratio = 1.96) (Table 5).

**Table 4:** Factors associated with the incidence of hair loss after contracting COVID-19. *: significant at level 0.05

Factor	Incidence of hair loss after contracting COVID-19		P-value
	Yes	No	
Age			0.003*
15–25 years	213 (64.7%)	116 (35.3%)	
26–35 years	117 (69.2%)	52 (30.8%)	
36–45 years	69 (63.3%)	40 (36.7%)	
46 years and older	26 (42.6%)	35 (57.4%)	
Gender			<0.001*
Male	118 (45%)	144 (55%)	
Female	307 (75.6%)	99 (24.4%)	
Marital status			0.165
Single	263 (65.9%)	136 (34.1%)	
Married	148 (59.2%)	102 (40.8%)	
Divorced	12 (80%)	3 (20%)	
Widowed	2 (50%)	2 (50%)	
Place of residence			0.092
Eastern region	108 (69.7%)	47 (30.3%)	
Central region	96 (65.3%)	51 (34.7%)	
Western region	103 (58.5%)	73 (41.5%)	
Southern region	65 (57%)	49 (43%)	
Northern region	53 (69.7%)	23 (30.3%)	
Q1: Did your temperature rise while you were infected with COVID-19?			0.002*
Yes	309 (67.5%)	149 (32.5%)	
No	116 (55.2%)	94 (44.8%)	
While you were infected with COVID-19, were you hospitalized?			0.633
Yes	47 (66.2%)	24 (33.8%)	
No	378 (63.3%)	219 (36.7%)	
Q2: During your COVID-19 infection, were you admitted to the intensive care unit?			0.876
Yes	4 (66.7%)	2 (33.3%)	
No	421 (63.6%)	241 (36.4%)	
Q3: Had you suffered from hair loss before contracting COVID-19?			<0.001*
Yes	225 (74.5%)	77 (25.5%)	
No	200 (54.6%)	166 (45.4%)	
Q4: Do you have a family history of alopecia?			0.091
Yes	87 (58.8%)	61 (41.2%)	
No	299 (66.4%)	151 (33.6%)	

**Table 5:** Multivariate logistic regression for factors predicting the incidence of hair loss after contracting COVID-19. *: significant at level 0.05

Factor	P-value	Odds ratio	Confidence interval	
Gender (male vs. female)	<0.001*	0.24	0.16	0.35
Age (15–25 years is the referent)				
26–35 years	0.028*	0.55	0.33	0.94
36–45 years	0.340	0.71	0.35	1.44
46 years and older	0.313	1.55	0.66	3.65
Marital status (single is the referent)				
Married	0.328	1.33	0.75	2.37
Divorced	0.408	0.50	0.10	2.61
Widowed	0.440	2.37	0.27	21.23
Place of Residence (the eastern region is the referent)				
Central region	0.480	1.23	0.70	2.17
Western region	0.002*	2.32	1.36	3.96
Southern region	0.019*	2.06	1.13	3.77
Northern region	0.320	1.43	0.71	2.91
Q1: Did your temperature rise while you were infected with COVID-19? (yes vs. no)	0.001*	1.96	1.31	2.93
Q2: While you were infected with COVID-19, were you hospitalized? (yes vs. no)	0.419	0.77	0.41	1.46
Q3: During your COVID-19 infection, were you admitted to the intensive care unit? (yes vs. no)	0.172	5.15	0.49	54.00
Q4: Do you have a family history of alopecia? (yes vs. no)	0.686	0.91	0.59	1.42

**Figure 1:**
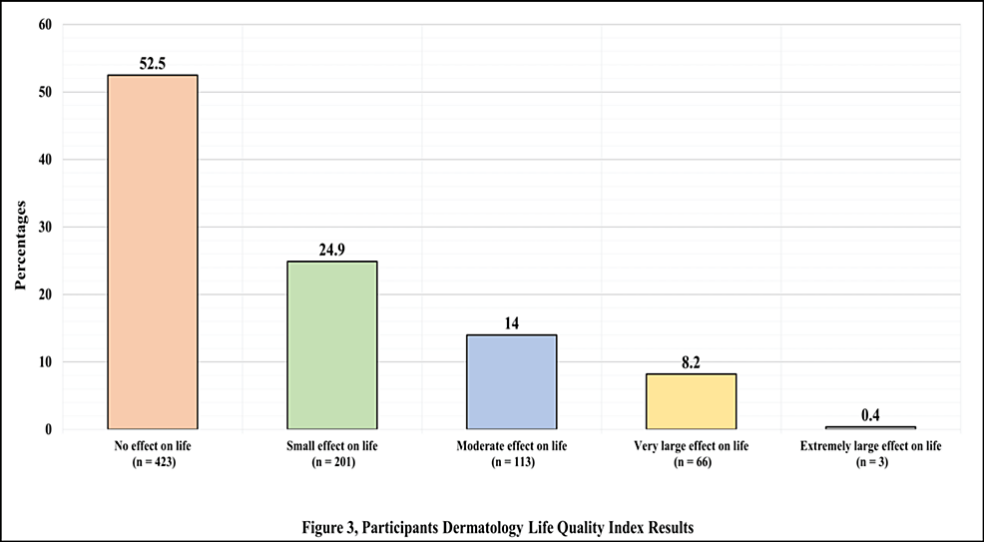
Participants’ Dermatology Life Quality Index results.

Of the participants, 423 (52.5%) reported that their quality of life was not affected at all. In contrast, only three (0.4%) reported that their hair loss had an extremely large effect on their life (Figure 1).

A significant difference in the mean of the DLQI score was observed when comparing those with hair loss and those without hair loss (p < 0.001) (4.84 ± 4.73 vs 0.99 ± 2.48) (Table 6).

**Table 6:** Comparison of the Dermatology Life Quality Index for those experiencing and not experiencing hair loss. *: significant at level 0.05

Factor	Dermatology Life Quality Index		P-value
	Mean	Standard deviation	
Have you suffered from hair loss after contracting COVID-19?			<0.001*
Yes	4.84	4.73	
No	0.99	2.48	

## Discussion

Hair shedding is a harrowing sequela of COVID-19. When infected with this virus, the increase in proinflammatory cytokines (tumor necrosis factor α, interleukin 1b, interleukin 6, and types 1 and 2 interferons) is believed to play a role in the development of hair shedding by damaging the hair follicle stem cell. However, the definite mechanisms of hair shedding following COVID-19 infection are not well known and further studies to elucidate the exact mechanisms are required [7]. Although hair loss is considered a benign condition, it can be devastating to a patient’s self-esteem, self-image, and overall quality of life.

Multiple studies have been conducted to investigate the development of TE as a consequence of COVID-19. Our study revealed that out of 806 participants, 425 (52.7%) experienced hair loss after contracting COVID-19. Similarly, a large study that investigated the prevalence and associated factors of COVID-19 hair loss among 5,891 patients found that hair loss was the most reported post-COVID-19 manifestation (2,800, 48%) [8]. According to the results of this study, hair loss after COVID-19 occurred earlier than did classic TE. A total of 196 (46.12%) participants noticed hair loss one to three months after having COVID-19. However, this interval is shorter than that observed with other febrile conditions in which hair loss is seen after three to four months from exposure to the triggering factor [9].

Our study also revealed that hair shedding was more common in women than men (75.5% vs. 54.6%). Moreno-Arrones et al. investigated 191 patients with TE who had been infected with COVID-19, 78.5% of whom were females [10]. TE is thought to be more common in females because they can undergo extreme stress, such as giving birth. Iron deficiency anemia is another trigger of this condition, which is more likely to occur in women due to menstruation. In addition, women take hair loss more seriously than men and therefore are more likely to consult a physician with this complaint [7].

In our series, we found that age was significantly associated with the incidence of hair loss post-COVID-19. Overall, 49.6% of those with hair loss were 15-25 years old, followed by 25.9% who were 26-35 years old, with the rest being 36-46 years old and older. Participants 46 years and older had a significantly lower rate of hair loss compared to other age groups. In contrast, a systematic meta-analysis of cohort studies found prolonged effects following acute COVID-19 in 80% of the study population aged 17-87 years. Because children are less commonly infected with SARS-CoV-2 and have less severe symptoms than adults with COVID-19, most hair loss cases appear at older ages [11]. In one study, 175 people were assessed for COVID-19. Of those with COVID-19 suffering from hair loss, the median age of the female patients was 71, and the median age of the male patients was 62.5 [12].

In our studied population, we found that 68.7% of participants experienced a high temperature during COVID-19 infection, while 31.3% did not. The incidence of hair loss after COVID-19 infection in people who reported a high temperature was 67.5%. Similarly, another study on 10 patients with COVID-19 reported that post-infection hair loss was associated with the severity of the disease and high-grade fever [13].

Only 13.41% of participants suffered from hair loss before contracting COVID-19, while 86.59% did not. A study done in Turkey revealed that 25.6% of the sample had TE, 4.4% had scalp alopecia areata (SAA), and 19.5% had seborrheic dermatitis (SD) before the pandemic, whereas 74.4%, 95.6%, and 80.5% did not have TE, SAA, or SD, respectively [12]. According to our study, the incidence of hair loss after COVID-19 in those who had previously experienced hair loss prior to COVID-19 was 74.5%, while 25.5% did not. A small number of studies have investigated the development of TE as a result of COVID-19. A study by Kutlu and Metin found that the incidence of TE increased 5.51 times during the COVID-19 pandemic compared to the same month the previous year, increasing from 0.40% to 2.17% [5]. Similarly, roughly three to four months after COVID-19 was declared a pandemic, Cline et al. found a rise in the incidence of TE from 0.5% to 2.3% [5]. However, in a study done at Istanbul Medeniyet University Prof. Dr. Suleyman Yalcin City Hospital, TE was found in 36.7% of the patients, of whom 27.9% were determined to have COVID-19-associated TE after excluding cases with other potential causes of TE or those who had experienced hair loss prior to COVID-19 [5].

The COVID-19 pandemic has had psychological effects on the general population. Increased psychological stress can have a positive impact on the progression of many skin conditions and can potentially lead to aggravating the underlying disease(s). This is especially true for hair loss, for which a wealth of literature reports can be due to psychological stress. Furthermore, hair loss can be stressful on its own, and that stress can exacerbate hair loss. This could be another cause of hair loss in persons who have recovered from COVID-19 [7,9].

Hair loss can have a negative effect on self-esteem for both men and women. Low self-esteem, in addition to anxiety and worry about hair shedding, can affect the quality of life of an individual. Based on the DLQI, our study found that 69 (8.6%) COVID-19 patients had their lives severely affected by hair loss, while (91.4%) did not, and a significant difference in the mean of the DLQI scores was observed when comparing those with hair loss and those without hair loss (p < 0.001) (4.84 ± 4.73 vs. 0.99 ± 2.48).

The authors of this study believe that this is the first study to report on the prevalence and impact of hair loss on the quality of life among COVID-19 survivors from different regions of Saudi Arabia. However, various limitations of the study should be considered. First, it is a cross-sectional study and thus it is not possible to infer causality between the study’s variables. Second, because the data collected was based on an online survey, it may have some weaknesses, such as recall bias.

## Conclusions

Patients with COVID-19 reported many sequels such as fatigue, headache, and dyspnea. Dermatological issues were one of the long-term effects of COVID-19 such as TE. This study revealed subjective evidence of hair loss in those who had contracted COVID-19 that was significantly associated with high body temperature, being female, and having a history of hair loss. Hence, physicians should be aware of the relationship between this infection and hair loss to improve patient health outcomes and their medical care. Although hair loss can have a dramatic effect on self-esteem and quality of life, the majority of the respondents reported that hair loss after COVID-19 did not affect their quality of life. Further studies using objective assessments of hair loss are suggested for reaching more precise conclusions.
